# Ketone bodies in kidney diseases: novel insights

**DOI:** 10.3389/fimmu.2026.1738916

**Published:** 2026-06-15

**Authors:** Wenni Dai, Weiying Zhang, Hao Li, Yang Gao, Li Xiao, Yinyin Chen, Liyu He

**Affiliations:** 1 Department of Nephrology, Hunan Key Lab of Kidney Disease and Blood Purification, and National Clinical Research Center for Metabolic Diseases, The Second Xiangya Hospital, Central South University, Changsha, Hunan, China; 2 Department of Nephrology, Hunan Provincial People’s Hospital, The First Affiliated Hospital of Hunan Normal University, Changsha, Hunan, China

**Keywords:** exogenous ketone supplement, HMGCS2, ketogenic diet, ketone body metabolism, SGLT2 inhibitor

## Abstract

Ketone bodies (KBs) are important energy fuels and play complex roles in the occurrence and development of diseases by regulating metabolism, inflammation, and multi-organ cellular crosstalk. The high mortality and morbidity of kidney diseases is an urgent global public health problem. The pathogenesis of kidney diseases is complex, with metabolic disorders underlying. Ketone body metabolism has recently become a hot spot, and ketogenic diets (KDs), sodium-glucose cotransporter 2 inhibitors (SGLT2i) along with exogenous ketone body supplements have drawn much attention for their role in treating obesity and type 2 diabetes. Therefore, it is essential to clarify the effects of KBs on kidney diseases to understand the occurrence and development of kidney diseases correctly and guide clinical treatment. This review aims to summarize the current findings on the impact of ketone body metabolism, KDs, SGLT2i and exogenous ketone body supplements on kidney diseases.

## 1 Introduction

Kidney diseases are a significant category of disorders affecting human health worldwide (1), with more than 850 million patients worldwide suffering from kidney diseases (2). According to the 2020 WHO report, chronic kidney disease (CKD) is one of the top 10 causes of death. By 2040, CKD is projected to become the fifth leading cause of death globally (3). Therefore, more researches on prevention, diagnosis, and treatment are needed to delay kidney diseases progression, improve therapeutic outcomes, and reduce mortality.

The kidney is one of the most energy-consuming organs. Its function relies on the constant production and efficient use of ATP. Impairments in energy production or utilization will lead to the occurrence and development of various kidney diseases (4). Under physiological conditions, renal ATP production primarily depends on mitochondrial oxidative metabolism, with fatty acid oxidation serving as the dominant energy source in proximal tubular cells, while glycolysis is more active in the renal medulla. In contrast, the energy produced by ketone bodies (KBs), lactic acid, and amino acids is minimal.

KBs are endogenous metabolites produced by the liver as a source of energy for peripheral tissues (5). KBs can provide energy to the body after prolonged fasting, severe carbohydrate restriction, or strenuous exercise. Under these extreme conditions, the liver produces up to 300g of KBs per day, accounting for approximately 5-20% of total energy expenditure (6). In addition to serving as energy substrates, KBs also act as signaling molecules to modulate cellular functions (7). Ketone body metabolism has been linked to cancer (8–11), obesity (12–14), diabetes (15–18), and cardiovascular disease (19–22). A growing body of research suggests that ketone body metabolism is also linked to kidney diseases, and elevated ketone body levels within certain ranges may be beneficial (23). However, excessively elevated circulating ketone body levels may damage the kidneys through unknown mechanisms (16). Notably, a recent study from the perspective of mammalian lifespan have further confirmed this dual nature: the loss of endogenous ketogenesis shortens lifespan, whereas unrestricted ketogenic diets (KDs) or excessive ketone body supplements early in life increases mortality, suggesting that the effects of KBs depend on the timing of intervention and the physiological state of the organism (24).

The KDs are very low-carbohydrate, high-fat and adequate-protein diet regimens that mimics the metabolism of the fasting state to induce the production of KBs. It began as a proven non-drug therapy for refractory epilepsy in children (25). In the field of neuroscience, studies have shown that diet-induced hyperketonemia can improve neurological disorders including Parkinson’s disease, Alzheimer’s disease, epilepsy, multiple sclerosis (26–30), as well as mental illnesses such as schizophrenia, bipolar affective disorder, symptoms of depressive disorders, and anxiety symptoms (31–33). Exogenous ketone body supplements can achieve mild ketosis without dietary restrictions. KDs and ketone body supplements are gaining popularity because they can be used to lose weight and improve glucose metabolism. Earlier studies have shown that short-term implementation of KDs can reduce weight effectively, even better than traditional low-fat diets (34). However, KDs often have significant side effects, including fatigue, gastrointestinal dysfunction, arrhythmia, constipation, diarrhea, headache, and stunting (12). In recent years, there has also been increasing research on KDs and ketone body supplements in kidney diseases, such as diabetic nephropathy (DN), acute kidney injury (AKI), autosomal dominant polycystic kidney disease (ADPKD), nephropathic cystinosis and so on (35–39).

This review focuses on the differential roles of renal ketone body metabolism in various kidney diseases and systematically explores the potential therapeutic value of ketosis for the kidney, aiming to provide a reference for future researches.

## 2 Ketone body metabolism

### 2.1 Overview of ketone body metabolism

**Figure 1:**
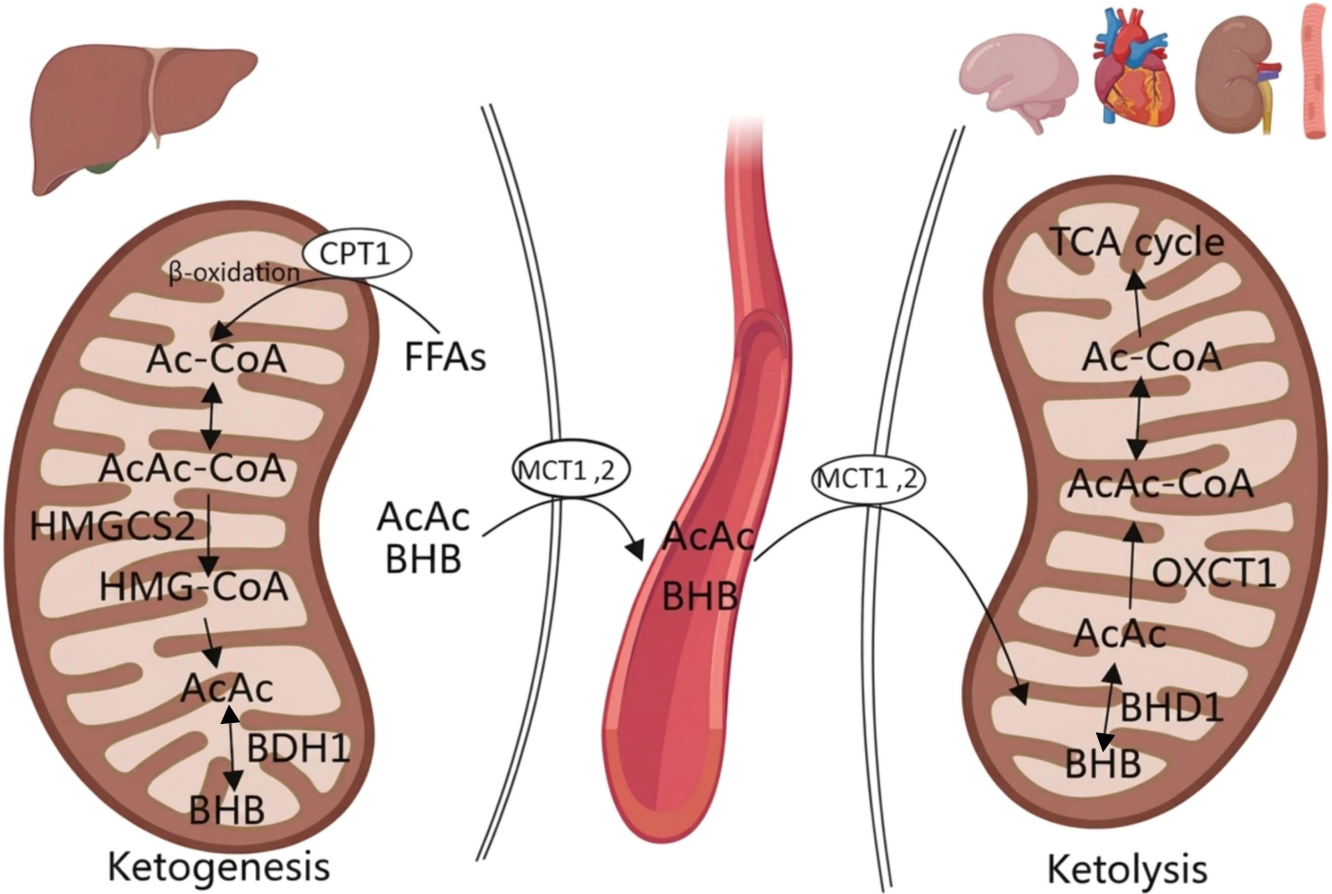
Overview of the ketogenesis and ketolysis pathways. Ketogenesis in hepatic mitochondria is the primary source of circulating KBs and requires using the key enzyme HMGCS2. KBs are secreted via MCT1/2, and their primary metabolic fate is terminal oxidation in extrahepatic tissue (such as the brain, heart, kidneys, and muscles) mitochondria via reactions that require the enzyme OXCT1. BHB and AcAc can be oxidized and converted to Ac-CoA, producing ATP via the TCA cycle as an alternative energy source. FFAs, free fatty acids; CPT1, carnitine palmityl transferase I; Ac-CoA, acetyl-coenzyme A; AcAc-CoA, acetoacetyl- coenzyme A; HMGCS2, hydroxy methylglutaryl-CoA synthase; AcAc, acetoacetate; BDH1, mitochondrial BHB dehydrogenase; BHB, β-hydroxybutyrate; MCT1/2, monocarboxylate transporter 1 and 2; OXCT1, 3-oxoacid CoA-transferase 1; TCA cycle, tricarboxylic acid cycle.

KBs include β-Hydroxybutyric acid (BHB, 78%), acetoacetate (AcAc, 20%), and acetone (2%), which can replace glucose to provide energy to the body during glucose deficiency. KBs’ metabolism includes ketogenesis and ketolysis (**Figure 1**). KBs are mainly synthesized in the mitochondria of hepatocytes from acetyl CoA produced by β-oxidation of fatty acids (40, 41). The mitochondria of hepatocytes contain various enzymes for synthesizing KBs, and 3-hydroxy-3-methylglutaryl-CoA synthase 2 (HMGCS2) is the critical enzyme for ketone body synthesis (42). Catalyzed by HMGCS2, acetoacetyl-CoA binds to a third acetyl-CoA molecule to form 3-hydroxy-3-methylglutaryl monoacetyl coenzyme A (HMG-CoA), which is cleaved by HMG-CoA cleavage enzyme (HMGCL) to form AcAc. AcAc, a precursor of BHB and acetone, is catalyzed by β-hydroxybutyrate dehydrogenase (BDH1) to produce BHB, which can also be decarboxylated spontaneously or catalyzed by acetoacetate decarboxylase to produce acetone (40). Hepatic-synthesized KBs are transferred into bloodstream via MCT1/2 and then used by extrahepatic organs such as the brain, heart, and skeletal muscle.

### 2.2 KBs as signaling molecules

For a long time, KBs have been regarded as alternative energy sources delivered by the liver to extrahepatic tissues during starvation and stress. However, accumulating evidences in recent years have demonstrated that KBs can act as signaling molecules to directly regulate cellular functions through multiple pathways. This part elaborates on the underlying mechanisms from four aspects: regulation of G protein-coupled receptors, regulation of histone deacetylases, remodeling of DNA methylation, mediation of β-Hydroxybutyrylation.

#### 2.2.1 Regulation of G protein-coupled receptors

G protein-coupled receptors (GPCRs) constitute the largest superfamily of membrane receptors and mediate signal transduction by activating G proteins. BHB acts as an endogenous ligand for hydroxycarboxylic acid receptor 2 (HCAR2), which is predominantly expressed in adipocytes, immune cells, and the kidney. Upon HCAR2 activation, BHB inhibits adenylyl cyclase (AC) via G proteins, thereby reducing intracellular cyclic adenosine monophosphate (cAMP) levels and triggering downstream signaling events. In immune cells, HCAR2 activation markedly suppresses the assembly and activation of the NOD-like receptor thermal protein domain associated protein 3 (NLRP3) inflammasome and decreases the maturation and secretion of pro-inflammatory cytokines such as interleukin-1β (IL-1β). Meanwhile, it attenuates the nuclear translocation and transcriptional activity of nuclear factor-κB (NF-κB), ultimately exerting anti-inflammatory effects (43–45). In adipocytes, HCAR2 activation inhibits hormone−sensitive lipase (HSL), reduces fatty acid release, and exerts an antilipolytic effect to maintain systemic lipid metabolic homeostasis (46–48). In addition, BHB can antagonize free fatty acid receptor 3 (FFAR3), which is mainly expressed in sympathetic ganglia. By inhibiting FFAR3 activity, BHB reduces sympathetic nerve tone and decreases the basal metabolic rate under starvation, thereby preserving energy reserves (49). AcAc serves as an endogenous ligand for free fatty acid receptor 2 (FFAR2) and plays a vital role in lipid metabolism and energy regulation (50).

#### 2.2.2 Regulation of histone deacetylases

Previous studies have demonstrated that BHB acts as an endogenous inhibitor of class I and IIa histone deacetylases (HDACs) (7). Elevated intracellular BHB levels, such as those induced by starvation, KDs or strenuous exercise, suppress HDAC activity and increase the acetylation levels of key histone sites including H3K9 and H3K14 (51, 52). HDAC inhibition remodels chromatin from a condensed to a relaxed state, thereby increasing the accessibility of transcription factors such as FOXO3a to target gene promoters. This further activates the transcription of downstream genes, including antioxidant enzymes (SOD2, catalase), metallothionein MT2 and so on (53, 54). Meanwhile, Shimazu T et al. found that BHB treatment did not significantly increase intracellular acetyl−CoA levels or enhance histone acetyltransferase (HAT) activity. This indicates that the BHB−induced elevation of histone acetylation is not primarily dependent on increased acetyl donor availability (54). However, recent studies have also reported that BHB exerts protective effects by activating HDAC5 in cisplatin−induced renal injury models. Therefore, further research is still required to fully clarify the regulatory effects of BHB on HDACs (55).

#### 2.2.3 Remodeling of DNA methylation

BHB can also indirectly regulate DNA methylation status through metabolic pathways, mainly via two mechanisms. First, KDs or BHB treatment elevates adenosine levels and reduces DNA methylation in the hippocampus (56). This may be attributed to the metabolic linkage between adenosine and S-adenosylhomocysteine (SAH). Because SAH functions as a natural inhibitor of DNA methyltransferase (DNMT), elevated adenosine levels suppress DNMT activity, thereby reducing global DNA methylation levels. In a chronic rat model of epilepsy, ketogenic dieting raises adenosine content and restores global DNA methylation, and such epigenetic alterations persist even after dietary intervention is discontinued (57). Second, a recent study has revealed that BHB inhibits the activity of S-adenosylhomocysteine hydrolase (AHCY) under ketogenic conditions. This disturbance impairs one-carbon metabolism and alters the abundance of related metabolites (58). Meanwhile, AHCY plays a critical role in maintaining SAH homeostasis. Thus, the above processes may further alter the SAM/SAH ratio and subsequently affect methylation status. Notably, direct experimental evidence supporting the regulation of DNA demethylases by BHB remains limited, and further investigations in this field are still warranted.

#### 2.2.4 Mediation of β-Hydroxybutyrylation

Independently, BHB itself directly modifies histone lysine residues (59). Under starvation or ketogenic conditions, intracellular BHB concentration increases and is converted into β-hydroxybutyryl-CoA. This metabolite is further catalyzed by acyltransferases including p300/CBP, which covalently attach β-hydroxybutyryl groups to lysine residues of target proteins (60–62). Kbhb modification exerts dual regulatory functions. At the histone level, Kbhb acts as a distinct epigenetic marker independent of acetylation. It is enriched in the promoter regions of metabolism−related genes, such as H3K9bhb, and regulates the transcription of genes involved in amino acid transport and gluconeogenesis (63, 64). At the non−histone level, multiple metabolic enzymes, including the key ketone metabolic enzyme 3-oxoacid CoA-transferase 1 (OXCT1), have been identified to undergo Kbhb modification (65), suggesting that this modification may modulate metabolic enzyme activity and ketone utilization. Kbhb modification is dynamically reversible, and its de-modification process is mediated by multiple deacylases. Current evidence indicates that members of the sirtuin family play a vital role in the removal of Kbhb marks (61). In addition, certain HDACs may also participate in Kbhb deacetylation (66); however, their substrate specificity and physiological relevance remain to be further elucidated.

## 3 Ketone body metabolism in the normal kidney

**Figure 2:**
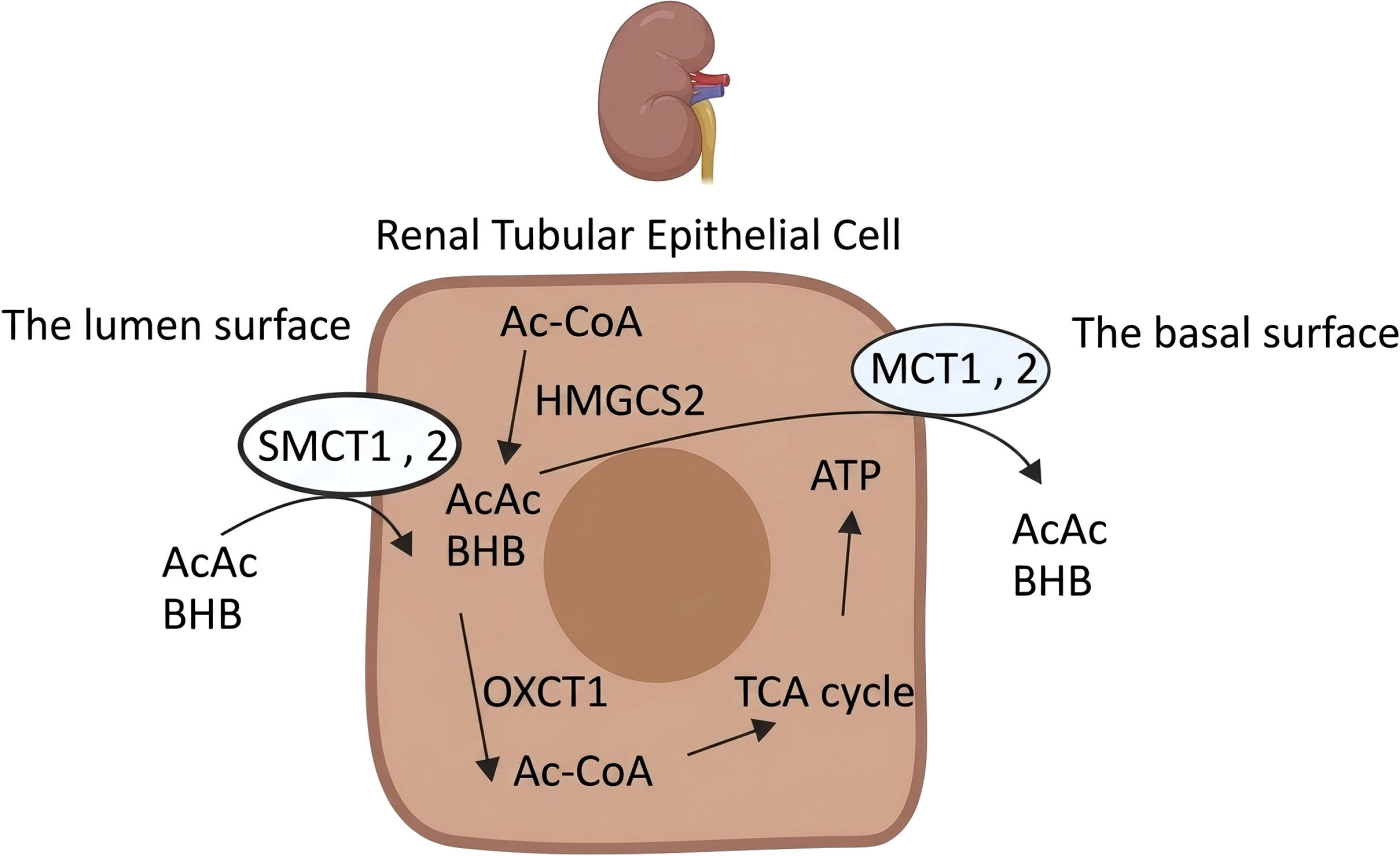
Overview of renal ketone body metabolism. The kidney performs multiple functions in ketone body metabolism, including reabsorption, oxidative utilization, and local synthesis. AcAc, acetoacetate; BHB, β-hydroxybutyrate; SMCT1/2, sodium-coupled monocarboxylate transporter 1 and 2; MCT1/2, monocarboxylate transporter 1 and 2; TCA cycle, tricarboxylic acid cycle; ATP, adenosine triphosphate.

Unlike the liver and brain, the kidney plays a unique role in ketone body metabolism. It is capable of both reabsorbing and utilizing KBs, as well as synthesizing them under specific conditions (**Figure 2**). These processes coordinately maintain renal energy homeostasis under diverse metabolic conditions.

### 3.1 Reabsorption of KBs

Circulating KBs predominantly BHB and AcAc, are filtered by the glomerulus and then actively reabsorbed by the proximal tubules (67). This process depends on luminal sodium-coupled monocarboxylate transporter 1 (SMCT1) and sodium-coupled monocarboxylate transporter 2 (SMCT2). Specifically, SMCT1 is mainly localized in the S2/S3 segments of the proximal tubule and possesses high affinity for KBs (68, 69). Following reabsorption, KBs can directly enter the mitochondria of renal tubular epithelial cells for oxidative energy supply. Alternatively, they re-enter the systemic circulation with the participation of basolateral monocarboxylate transporter 1 (MCT1) and monocarboxylate transporter 2 (MCT2) (70). Under fasting conditions, the renal reabsorption capacity for KBs is markedly enhanced, highlighting the crucial role of the kidney in maintaining whole-body energy homeostasis (71).

### 3.2 Oxidative utilization of KBs

OXCT1, a key enzyme that breaks down KDs, is expressed in the mitochondria of renal tubules. It catalyzes the conversion of AcAc into acetoacetyl-CoA, which is subsequently cleaved by thiolase to yield two molecules of acetyl-CoA. Acetyl-CoA then enters the tricarboxylic acid cycle to generate ATP. The activity of OXCT1 is highly correlated with mitochondrial density, with the highest levels detected in the thick ascending limb and distal convoluted tubule (72, 73). Studies have demonstrated that AcAc can supply up to 80% of the energy required by the normal kidney, underscoring the central role of ketone body oxidation in renal energy metabolism (67).

### 3.3 Renal synthesis of KBs

Traditionally, ketogenesis is thought to occur mainly in liver mitochondria, where it is regulated by the rate-limiting enzyme HMGCS2. However, emerging evidence indicates that normal kidneys also express HMGCS2, and its expression in the renal cortex is markedly upregulated under physiological metabolic stress conditions such as fasting or a ketogenic diet (74–76). Mechanistically, the ketogenic diet-induced upregulation of renal HMGCS2 is associated with enhanced PPAR-α activation (74). Experiments in renal proximal tubule cells further demonstrated that PPAR-α activation increases HMGCS2 expression, whereas PPAR-α knockdown abolishes this effect, identifying PPAR-α as an upstream transcription factor regulating renal HMGCS2 expression (77). Despite the ketogenic capacity of the kidney, its contribution to circulating ketone bodies is limited. Liver-specific HMGCS2 knockout dramatically reduces circulating ketone levels in mice. In contrast, kidney-derived ketone bodies are largely confined to local metabolism and do not significantly affect systemic ketone concentrations, suggesting that renal ketogenesis primarily serves local metabolic demands (75). Moreover, a ketogenic diet also markedly upregulates the expression of BDH1 and MCT1 in normal kidneys, further enhancing renal ketone uptake and utilization. Together, these findings illustrate the adaptive capacity of renal metabolism in response to ketogenic diet intervention (74).

## 4 The role of ketone body metabolism in kidney diseases

**Figure 3:**
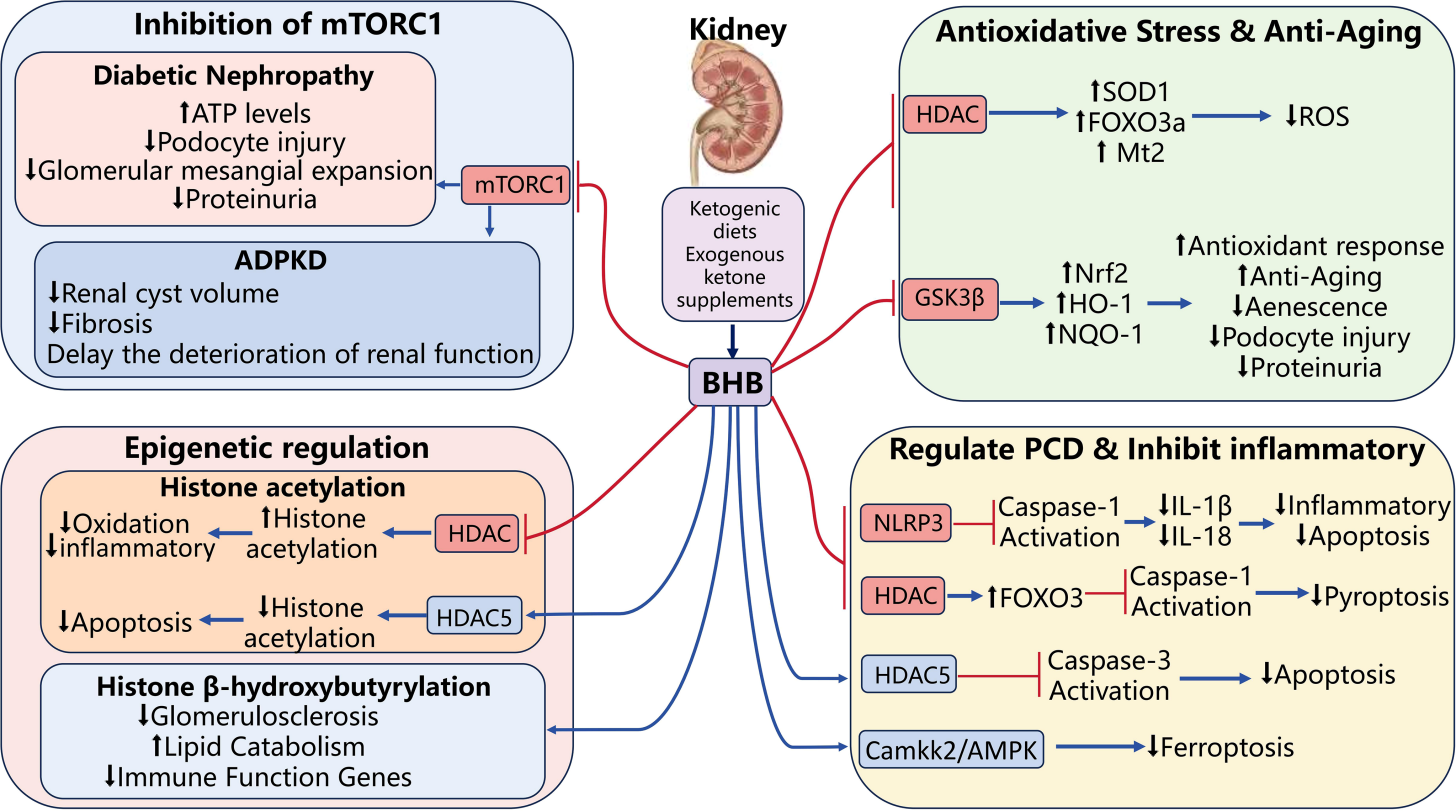
Schematic diagram of the renoprotective mechanisms of KBs. KBs (primarily BHB) effectively alleviate podocyte injury, renal fibrosis and renal function deterioration in pathological conditions such as DN and ADPKD. These protective effects are achieved through the inhibition of mTORC1, anti-oxidative stress, epigenetic regulation, and suppression of programmed cell death, thereby endowing KBs with prominent renoprotective potential. Arrows indicate activation, and truncated lines indicate inhibition. ADPKD, Autosomal Dominant Polycystic Kidney Disease; AMPK, AMP-activated Protein Kinase; BHB, β-Hydroxybutyrate; Camkk2, Calcium/Calmodulin-dependent Protein Kinase Kinase 2; FOXO3, Forkhead Box O3; GSK3β, Glycogen Synthase Kinase 3β; HDAC, Histone Deacetylase; HO-1, Heme Oxygenase-1; IL. Interleukin; mTORC1, Mammalian Target of Rapamycin Complex 1; Mt2, Metallothionein 2 (mouse gene); NLRP3, NLR Family Pyrin Domain-containing 3; NQO1, NAD(P)H Quinone Dehydrogenase 1 (mouse gene); Nrf2, Nuclear Factor Erythroid 2-related Factor 2; PCD, Programmed Cell Death; ROS, Reactive Oxygen Species; SOD1, Superoxide Dismutase 1.

Ketone body metabolism exhibits complex dual effects in kidney diseases. On the one hand, BHB exerts renoprotective functions through multiple signaling pathways (**Figure 3**). On the other hand, under specific pathological conditions such as hyperglycemia, abnormal alterations in HMGCS2 may drive disease progression.

### 4.1 Renoprotective mechanisms of KBs

#### 4.1.1 Inhibition of the mTORC1 signaling pathway

The Ser/Thr kinase mechanistic target of rapamycin (mTOR) serves as a core regulator of cellular metabolism. The mTOR complex 1 (mTORC1) signaling pathway acts as a critical regulatory hub that integrates cell growth, metabolic reprogramming, protein synthesis, and autophagy. Its excessive activation contributes to podocyte hypertrophy, abnormal proliferation of renal tubular epithelial cells, and excessive extracellular matrix deposition, which constitute a common pathogenic mechanism underlying DN and polycystic kidney disease (78). In DN models, BHB restores renal tubular ATP levels, alleviates podocyte injury and glomerular mesangial expansion, and reduces proteinuria by directly suppressing aberrant mTORC1 overactivation (79). The renoprotective effects mediated by odium-glucose cotransporter 2 inhibitors (SGLT2i) are partially dependent on enhanced endogenous ketone production. Notably, such protective effects are markedly reversed by HMGCS2 knockout, further validating the essential role of the KBs–mTORC1 axis in maintaining renal homeostasis (80). In polycystic kidney disease models, interventions with KDs or exogenous ketone supplements retard renal cyst expansion, mitigate fibrosis, and slow renal function decline via mTORC1 inhibition (81).

#### 4.1.2 Anti-oxidative stress and anti-aging effects

Oxidative stress and cellular senescence are recognized as the core pathological foundations of kidney diseases, particularly in DN. Excessive accumulation of reactive oxygen species (ROS) triggers DNA damage, lipid peroxidation and mitochondrial dysfunction, thereby driving renal tubular epithelial cell apoptosis and interstitial inflammation (82, 83). In DN mouse models, KDs have been proven to reduce proteinuria and downregulate the expression of stress- and toxicity-related genes (84).

As the major metabolic product of KDs, BHB exerts prominent renoprotective effects. Acting as an endogenous class I HDAC inhibitor, BHB upregulates the transcription of antioxidant genes such as SOD1, FOXO3a and MT2, and consequently reduces ROS accumulation in renal tubular epithelial cells and podocytes (54). Studies have demonstrated that high glucose and TGF-β1 induce excessive GSK3β activation in podocytes under DN conditions. BHB directly binds to the ATP-binding pocket of GSK3β and inhibits its kinase activity. This blockade suppresses GSK3β-mediated phosphorylation of the Nrf2 protein at the Ser40 site, prevents Nrf2 nuclear export and degradation, and promotes nuclear accumulation of Nrf2 to facilitate the transcription of downstream antioxidant genes including HO-1 and NQO1. This mechanism strengthens the antioxidant defense capacity of podocytes, alleviates cellular senescence and injury, and ultimately ameliorates proteinuria (85).

#### 4.1.3 Epigenetic regulation

In recent years, BHB has been confirmed to participate in the pathophysiological regulation of kidney diseases through multiple epigenetic modification mechanisms, mainly involving histone acetylation and lysine Kbhb.

At the level of histone acetylation, BHB exhibits bidirectional regulatory effects. On the one hand, as an endogenous class I HDAC inhibitor, BHB increases the acetylation levels of histone residues such as H3K9 and H3K14, thereby activating the expression of genes associated with antioxidation, anti-inflammation, and mitochondrial function (53, 54). For example, in renal ischemia-reperfusion injury, BHB inhibits pyroptosis by restoring the acetylation level of the FOXO3 promoter region (86). On the other hand, in cisplatin-induced AKI models, BHB exerts protective effects by specifically activating HDAC5 (Class II HDAC), which markedly inhibits Caspase-3 activation and reduces apoptosis in human renal cortical epithelial (HRCE) cells (55). Such seemingly contradictory bidirectional regulation indicates that BHB acts selectively on distinct members of the HDAC family, and its final biological effects depend on the injury type and cellular context.

At the level of histone Kbhb, BHB acts as a direct substrate to mediate the regulation of this novel epigenetic modification. In DN, BHB enhances H3K9bhb modification in the promoter region of Mmp-2, thereby antagonizing glomerulosclerosis (87). In hypertensive renal injury, BHB-mediated Kbhb modification remodels chromatin structure, upregulates the expression of lipolytic genes such as HMGCS2, and suppresses the transcription of immune genes, thereby achieving coordinated regulation of metabolism and immunity (88).

#### 4.1.4 Inhibition of inflammatory and regulation of programmed cell death

BHB also exerts renoprotective effects by suppressing inflammatory responses and inhibiting apoptosis, pyroptosis, and ferroptosis. In cisplatin-induced AKI mouse models and human renal tubular epithelial cells (HK-2), BHB blocks the activation of the NLRP3 inflammasome and reduces the Caspase-1-dependent maturation and release of pro-inflammatory cytokines such as IL-1β and IL-18, thereby alleviating renal inflammation and attenuating cellular apoptosis (89). Further studies have revealed that BHB inhibits Caspase-3 activation by activating HDAC5, thereby reducing apoptosis in HRCE cells during cisplatin-induced AKI (55). In addition, BHB can restore histone acetylation levels in the FOXO3 promoter region by inhibiting HDAC activity, thereby upregulating FOXO3 expression in renal tubular epithelial cells. This suppresses Caspase-1 activation and the release of downstream inflammatory factors, ultimately blocking the pyroptosis signaling pathway (86). Notably, emerging studies have further expanded our understanding of BHB-mediated cell death regulation. In cisplatin-induced AKI models, BHB also inhibits ferroptosis in renal tubular epithelial cells and improves mitochondrial function by activating the Camkk2/AMPK signaling pathway, ultimately alleviating renal damage (90). In summary, BHB establishes a multi-layered renoprotective network by coordinately modulating inflammatory responses and multiple cell fate pathways, including apoptosis, pyroptosis, and ferroptosis. These findings provide novel therapeutic targets for ketone-based intervention in kidney diseases.

### 4.2 HMGCS2-mediated dual roles: from renoprotection to pathogenicity

HMGCS2 is the key rate-limiting enzyme for ketogenesis. Accumulating studies have revealed its vital roles in kidney diseases (79, 91). Overall, HMGCS2 predominantly exerts protective effects in AKI. In contrast, its sustained abnormal activation and specific post-translational modifications may drive disease progression in DN and certain CKD models (92, 93).

#### 4.2.1 Protective effects of HMGCS2 in AKI

HMGCS2 exerts a protective role in AKI. Feola et al. demonstrated using renal tubule-specific HMGCS2 knockout mice that HMGCS2 deficiency impairs renal ketogenesis, severely compromises mitochondrial fatty acid oxidation, and causes excessive lipid accumulation, thereby markedly exacerbating ischemia-reperfusion-induced tubular injury. Preconditioning with KDs restore fatty acid oxidation and attenuates renal damage, confirming the renoprotective significance of local renal ketogenesis in AKI (94). In terms of regulatory mechanisms, two studies have uncovered the sophisticated regulatory network of HMGCS2 at the transcriptional and post-translational levels respectively. One study has been found that the extracellular matrix component MFAP2 modulates HMGCS2 expression via ESR2 at the transcriptional level, and MFAP2 deficiency can aggravate AKI (95). Another study has shown that Calponin 2 is upregulated after AKI and negatively regulates the activity of the desuccinylase Sirtuin 5. Knockdown of Calponin 2 activates Sirtuin 5, which further mediates the desuccinylation modification of HMGCS2 to enhance its activity, promote ketone production and ATP generation, and thereby alleviate renal injury (96). Although HMGCS2 exerts protective effects in ischemic AKI, its expression is abnormally elevated and closely associated with renal injury in diquat-induced nephrotoxicity, indicating that the pathological roles of HMGCS2 are dependent on toxic stress conditions. Nevertheless, whether such upregulation serves merely as a biomarker of damage or acts as a direct pathogenic driver remains to be verified by further functional experiments (97).

#### 4.2.2 Pathogenic role of HMGCS2 in DN

In the diabetic state, the kidney has been identified as an active ketogenic organ (98). HMGCS2 is markedly upregulated in the kidneys of both type 1 and type 2 diabetic mice and exerts a pathogenic role in DN (99, 100). In podocytes, fructose enhances fatty acid degradation by increasing HMGCS2 expression, thereby disrupting mitochondrial structure and function and exacerbating cellular injury (91). In renal endothelial cells, aberrant HMGCS2 activation also triggers mitochondrial dysfunction and pyroptosis (101). Moreover, SGLT2i alleviates renal fibrosis in DN by suppressing HMGCS2 expression in proximal tubular cells (92). Emerging evidence further reveals that HMGCS2 undergoes desuccinylation in renal tubules under diabetic conditions, which elevates its enzymatic activity and leads to excessive AcAc production. As a paracrine mediator, AcAc binds to the macrophage surface receptor GPR109A, promotes M1 macrophage polarization and pro-inflammatory cytokine secretion, and ultimately constructs a maladaptive tubule–macrophage inflammatory crosstalk loop to aggravate tubular damage and renal fibrosis (93). Collectively, these findings demonstrate that HMGCS2 drives DN progression through a multicellular and multi-mechanistic pathogenic network. Targeting HMGCS2 and its downstream metabolic-immune axis holds great therapeutic potential for DN treatment.

#### 4.2.3 Diverse functions of HMGCS2 in CKD

HMGCS2 exhibits markedly context-dependent functional effects in CKD. Its expression patterns and pathological roles vary greatly across different etiologies and experimental models. Studies have shown that the downregulation of the mitochondrial protease LONP1 causes abnormal accumulation of its substrate HMGCS2 within mitochondria, thereby impairing mitochondrial function and accelerating CKD progression (102). Triclosan downregulates HMGCS2 expression by inhibiting the PPARα signaling pathway, resulting in impaired local renal ketogenesis, aberrant lipid accumulation, and mitochondrial dysfunction, which ultimately exacerbate renal fibrosis. In contrast, HMGCS2 overexpression maintains mitochondrial homeostasis and alleviates renal fibrosis (103). In addition, relevant studies have demonstrated that HMGCS2 partially contributes to antioxidant protection in the kidneys of stroke-prone spontaneously hypertensive rats (104). This suggests that the homeostasis maintenance of HMGCS2 is of universal significance for the defense against CKD; however, its specific regulatory mechanisms and intervention strategies should be differentiated according to distinct disease etiologies.

#### 4.2.4 Protective effects of HMGCS2 in renal cell carcinoma

The tumor-suppressive role of HMGCS2 in clear cell renal cell carcinoma (ccRCC) has been preliminarily elucidated. Clinical evidence indicates that HMGCS2 is markedly downregulated in tumor tissues, and its low expression is closely correlated with poor patient prognosis and dysregulated immune microenvironment (105). Functional experiments further confirmed that restoring HMGCS2 expression inhibits renal cell carcinoma proliferation and induces cell apoptosis (105). At the molecular level, hypermethylation occurs in the promoter region of the HMGCS2 gene in renal carcinoma cells. Meanwhile, abnormal m^6^A methylation modification of the upstream ACSM3 gene leads to its downregulation, thereby inhibiting the transcription of HMGCS2, Mechanistically, METTL14-mediated m^6^A methylation modulates ACSM3 expression, and ACSM3 in turn upregulates HMGCS2 via GATA5. This axis further suppresses the mTORC1 signaling pathway and reduces aberrant lipid accumulation, ultimately exerting tumor-suppressive effects (106). Collectively, these findings suggest that HMGCS2 serves as a promising diagnostic biomarker and therapeutic target for ccRCC.

### 4.3 Renoprotective roles of OXCT1 and SMCT1 in kidney diseases

The renoprotective effects of KBs rely not only on their HMGCS2-dependent biosynthesis but also on their catabolism and reabsorption. In adenine-induced renal injury models, exogenous ketones (e.g., 1,3-butanediol) markedly ameliorate renal fibrosis, inflammation and cellular apoptosis. However, such protective effects are greatly blunted in kidney-specific OXCT1 knockout mice, confirming that the renal protective actions of KBs depend on their catabolism and utilization mediated by OXCT1 (107). Meanwhile, as a critical transporter responsible for renal reabsorption of KBs (especially BHB), SMCT1 is significantly downregulated in patients with DN and corresponding mouse models. This reduction leads to decreased local renal KBs levels and ATP deficiency. In contrast, upregulation of SMCT1 restores mitochondrial energy metabolism and alleviates microalbuminuria (108). Collectively, these findings demonstrate that OXCT1-mediated ketone catabolism acts synergistically with SMCT1-dependent ketone reabsorption, jointly forming the core mechanism by which KBs exert renoprotective functions.

## 5 Ketosis-based strategies for kidney diseases

As mentioned above, ketone body metabolism plays a dual role in kidney diseases, exhibiting both protective and pathogenic effects. Precisely regulating ketone body metabolism to maximize benefits while minimizing harm has become a key focus for therapeutic translation. The main strategies currently include KDs, SGLT2i, and exogenous ketone supplements. These three strategies exert different effects in kidney diseases (**Table 1**).

**Table 1:** Summary of the ketosis strategy in kidney diseases.

Ketosis strategy	Models	Formulation/ dose	Duration	Effects & mechanisms	Bood BHB levels(mM)	Detection methods	References
KD	Akita and db/db mice	5% carbohydrate, 8% protein, 87% fat	8 weeks	Reversed renal stress- and toxicity-related gene expression	Akita-KD: 2.34 ± 0.3 (vs con 0.47) db/db-KD: 1.8 ± 0.5 (vs con 0.4) WT-KD: 0.63-1.1	the Precision Xtra Ketone Monitoring System (Abbott Laboratories. Abbott Park, IL).	(84)
1,3-butanediol	HFD-fed ApoE-/- mice	20% 1,3-BD in diet	8 weeks	Ameliorated tubular injury, fibrosis, inflammation; restored ATP; inhibited mTORC1	Treatment: ~1.3-1.5 Control: <0.1	the Ketometer (Abbott, Chicago, IL, USA)	(79)
	db/db mice		12 weeks	Reduced albuminuria; ameliorated podocyte injury; inhibited mTORC1	Treatment: ~0.20-0.28 (gradually increased over time) Control: ~0.08-0.10		
	Kidney-specific Tsc1 knockout mice		4 weeks	Inhibited mTORC1; reduced kidney injury and fibrosis; restored ATP; improved survival rate	Treatment: ~0.8-1.0 Control: ~0.05-0.10		
Empagliflozin + 8h fasting	HFD-fed ApoE-/- mice	30 mg/kg/day, oral gavage at 5:00 PM, fasting 9:00 AM-5:00 PM	8 weeks	Ameliorated tubular injury, fibrosis, inflammation; restored ATP; inhibited mTORC1	Treatment: ~0.35-0.40 Control: <0.05		
	db/db mice		12 weeks	Reduced albuminuria; ameliorated podocyte injury; inhibited mTORC1	not reported		
	Kidney-specific Tsc1 knockout mice		4 weeks	Inhibited mTORC1; reduced kidney injury; restored ATP; improved survival rate	Treatment: ~0.40 Control: ~0.05		
Exogenous supplements	STZ (streptozotocin)-induced type 1 diabetic C57BL/6 mice	i.p. 100 mg/kg/day	4 weeks	Reduced podocyte senescence/injury; Inhibited GSK3β, activated Nrf2; Ameliorated albuminuria	Increased within 2 hours after first dose, sustained for 16 hours (exact concentration not reported)	–	(85)
Exogenous supplements	STZ induced DN rats	s.c. 160, 200, and 240 mg/kg/day	10 weeks	Againsted diabetic glomerulosclerosis	Not measured	–	(87)
KD	Renal IRI model in rats	1.5% carbohydrate, 11.3% protein, 87.2% fat	3 days before surgery	Anti-inflammatory, Antioxidation	~0.85(vs con~0.15)	The β-hydroxybutyrate assay kit (Cayman Chemicals, Ann Arbor, MI, USA)	(113)
Preoperative fasting (2 days)	Mouse renal IRI model (C57BL/6J, 45 min ischemia, right nephrectomy)	48h fasting before surgery, free access to water	48h before → before surgery	Improved renal functions; Reduced tubular injury score; Inhibited pyroptosis Upregulated FOXO3; restored H3K9 acetylation	~2.0	the FreeStyle Precision Neo (Abbott, Lake Bluff, IL)	(86)
Exogenous BHB continuous infusion		Intraperitoneal osmotic pump (ALZET 2001D) BHB: 1 g/ml in PBS, 8 μl/h	24h before → 24h after surgery (48h total)		1.3–1.40 (maintained until 24h after surgery)		
BHB pre-infusion (stopped at surgery		Osmotic pump BHB infusion, stopped at time of IR surgery	Only 24h before surgery (no infusion after surgery)	Renal protection persisted at 24 h post-surgery despite BHB levels returning to baseline	- Pre-surgery (at stop): ~1.4 - 24 h post-IRI: ~0.35		
Fasting + BHB infusion		48h fasting + osmotic pump BHB infusion	48h fasting + infusion until surgery	No additive protection; combined treatment did not further attenuate renal injury	At surgery start: 4.73 mmol/L 24h after surgery: ~2.0 mmol/L		
Exogenous supplements	Cisplatin-induced AKI	s.c. 250 mg/kg/day	3 days after the injection of cisplatin	Inhibited NLRP3 inflammasome and oxidative stress	Not measured	–	(89)
Exogenous supplements	Cisplatin-induced AKI	s.c. 500 mg/kg/day	3 days after the injection of cisplatin	Anti-apoptosis via activation of HDAC5	Not measured	–	(55)
KD	Cisplatin-induced AKI	11% fat, 22% protein, and 67% carbohydrate in kcal	eight consecutive days	Attenuated ferroptosis via regulating the Camkk2-AMPK pathway.	0.3-1.5	The enzyme-linked immunosorbent assay (ELISA) kit (E-BC-K785-M, Elabscience, China)	(90)
Exogenous supplements		i.p.10 mmol/kg			1-2		
Time-Restricted Feeding (TRF)	Han: SPRD rats	8-hour daily access to food (dark cycle); normal chow (62% carb, 25% protein, 13% fat); water ad libitum	5 weeks (3-8w)	Reduced cyst burden Partially improved renal function (serum Cr ↓) Inhibited mTORC1/STAT3 Reduced fibrosis	0.4-0.5	the blood ketone meter (Abbott; Precision Xtra).	(81)
Ketogenic Diet (KD, juvenile)	Han: SPRD rats (3–8 weeks of age)	KD chow: 91% fat, 2% carb	5 weeks	Markedly reduced cyst burden Improved renal function Inhibited mTORC1/STAT3 Activated AMPK Reduced fibrosis	1.5-2.5		
Ketogenic Diet (KD, adult)	Han: SPRD rats (8–12 weeks of age)	KD chow; ad libitum	4 weeks	Reduced cyst size (but not cyst number or kidney/body weight ratio) Reduced fibrosis (males only) No effect on serum creatinine	1.5-2.0		
Acute Fasting (24h)	Pkd1 mouse	Complete fasting for 24 hours	24 hours	Induced apoptosis No significant effect on cyst volume	1.5-2.0		
Acute Fasting (48h)	Han: SPRD rat	Complete fasting for 48 hours	48 hours	~20% reduction in cystic index Induced apoptosis Cyst fluid loss Lipid accumulation	2.5-3.0		
Acute Fasting (72h)	PKD cat	Complete fasting for 72 hours	72 hours	15% mean reduction in total kidney volume	Not measured		
Oral BHB	Han: SPRD rat	BHB salt in drinking water (157.5 mM); normal chow	5 weeks (3-8w)	Cyst burden markedly reduced Significantly reduced fibrosis Myofibroblasts nearly eliminated Inhibited proliferation Improved renal function	Not measured		
High-fat ketogenic diet	Eker rats (Tuberous Sclerosis model)	79% fat, 0.8% carbohydrate, 9.5% protein, ad libitum	4 months (KD4) 6 months (KD6) 8 months (KD8)	KD4/KD6: No statistically significant difference with control KD8:Significantly increased tumor volume via activating ERK1/2 and mTOR pathways	Con: 0.68 ± 0.1 KD4: 3.2 ± 0.2 KD6: 3.8 ± 0.2 KD8: 3.4 ± 0.4	the β-Hydroxybutyrate Assay Kit (BioVision, Milpitas, CA, USA)	(116)
KD	Renal cell carcinoma mice	long-chain fatty acid KD (LCT); 25% 8-carbon medium-chain fatty acids and 49.6% LCT KD (LCT/MCT8); and 25% 10-carbon medium-chain fatty acids and 49.6% LCT KD (LCT/MCT10)	40 days	Reduced tumor growth but also overall survival of tumor bearing mice	elevated, but specific values were not provided.	the specific enzyme-based kit (Precision Xceed, Abbott Laboratories, Austria).	(117)

### 5.1 Ketogenic diets

Depending on nutritional composition, KDs can be classified into several categories. The classic KDs adopts a strict ratio of fat to combined carbohydrates and protein, typically at 4:1 (109). The modified Atkins diet imposes relatively lenient restrictions on carbohydrates and protein, leading to better patient compliance (110). The medium-chain triglyceride diet achieves comparable ketosis under moderate carbohydrate restriction through supplementary medium-chain triglycerides (111). The low-glycemic index therapy permits an appropriate intake of low-GI carbohydrates, representing the most flexible pattern and being closest to regular daily diets (110). In addition, intermittent fasting, including time-restricted feeding and alternate-day fasting, elevates circulating KBs levels by periodically prolonging the fasting duration, and is regarded in several studies as an alternative or adjuvant strategy to KDs.

KDs remodel systemic energy metabolism by inducing ketogenesis and exhibit promising therapeutic potential in a variety of kidney diseases (112). This section mainly summarizes the research advances in DN, AKI, ADPKD, renal tumors, and obesity-associated CKD.

Studies have demonstrated that KDs intervention can reverse pathological alterations caused by diabetic nephropathy in mouse models, especially in terms of the albumin−to−creatinine ratio and the expression of stress and toxicity−related genes (84). Clinical trials have indicated that KDs are correlated with a lower incidence of end−stage renal disease in patients with DN (36). These lines of evidence collectively suggest that KDs exert renoprotective effects against DN.

Accumulating evidence indicates that KDs exert prominent protective effects against AKI. In cisplatin-induced and ischemic AKI models, KDs elevate endogenous KBs levels, thereby reducing oxidative stress and inflammatory responses and alleviating overall acute renal damage (90, 113). In terms of the progression from AKI to CKD, KDs continuously suppress inflammation and oxidative stress, attenuate renal interstitial fibrosis and collagen deposition, and block the cascading “injury-inflammation-fibrosis” process (113).

Multiple animal models have demonstrated that dietary restriction and KDs effectively inhibit mTOR signaling, cell proliferation and fibrosis in affected kidneys, while reducing renal cyst burden (81). In recent years, several clinical studies have further suggested that KDs are well feasible in patients with ADPKD. Such interventions can improve body weight, blood pressure and partial metabolic parameters, and may exert beneficial effects on renal function and the enlargement of total kidney volume (38, 114, 115). Nevertheless, current clinical studies are limited by small sample sizes and short follow-up durations, with a lack of hard endpoint evidence. Meanwhile, concerns including elevated blood lipids, increased nephrolithiasis risk and long-term dietary adherence remain to be fully evaluated (38).

In terms of renal tumors, studies have found that long-term KDs for 8 months significantly accelerate renal tumor growth in tuberous sclerosis complex (TSC) rats, with a 12-fold increase in median tumor volume (p = 0.018). The serum BHB concentration ranges from 3.0 to 3.8 mM, accompanied by the activation of the ERK1/2/mTOR signaling pathway (116). Another study demonstrated that although KDs suppress the progression of renal cell carcinoma, they aggravate hepatic inflammation, induce body weight loss, and reduce the survival rate in tumor-bearing mice, while exerting no such adverse impacts on healthy counterparts (117). These findings indicate that KDs may pose potential risks of tumor progression and aggravated systemic injury, and their application should be carefully assessed in clinical practice.

Obesity is a major independent risk factor for CKD, which drives the onset and progression of CKD through multiple mechanisms including glomerular hyperfiltration and adipokine imbalance (118). As an effective weight-loss strategy, KDs can significantly reduce body weight and improve insulin sensitivity in the short term. Recent randomized controlled trials have demonstrated that, in obese patients with early CKD, KDs yield superior weight loss outcomes compared with conventional low-calorie diets, with no obvious deterioration of renal function observed within a short period (119–121). Notably, emerging studies have shown that Semaglutide reduces the urinary albumin-to-creatinine ratio (UACR), suggesting that weight loss-related renal benefits are not entirely dependent on KDs (122).

Long-term KDs may increase the risks of nephrolithiasis, acidosis, dyslipidemia, and electrolyte disorders. Nephrolithiasis has been reported in patients with ADPKD (38). For individuals with severely impaired renal function, KDs may trigger metabolic acidosis (123). KDs tend to elevate cholesterol and LDL-C levels (124), thereby requiring regular lipid monitoring. In the early stage of KD intervention, reduced insulin secretion and ketone-induced osmotic diuresis may lead to electrolyte disturbances such as hyponatremia, hypokalemia, and hypomagnesemia (125).

At present, the clinical application of KDs in kidney diseases is mainly limited to patients with ADPKD and obesity-associated CKD. Further experimental and clinical evidence is still required to explore its efficacy in other kidney disorders.

### 5.2 SGLT2 inhibitors

SGLT2i reduces glucose reabsorption in the proximal tubule and promote urinary glucose excretion. Meanwhile, they shift systemic substrate utilization toward fatty acid oxidation and mild ketogenesis, thereby moderately increasing circulating BHB levels (126–128). In mouse models of DN, SGLT2i inhibits the overactivated renal mTORC1 pathway and restore autophagic function by elevating circulating BHB, which further alleviates glomerular hypertrophy, podocyte injury and proteinuria (79). Studies have demonstrated that in diabetic patients with cardiovascular diseases, SGLT2i suppresses the assembly and activation of the NLRP3 inflammasome via KBs, and reduce the maturation and release of pro-inflammatory cytokines (129). Multiple randomized controlled trials have confirmed that in both diabetic and non-diabetic CKD populations, these drugs reduce the risks of renal failure and renal or cardiovascular mortality by 25%–40% (130).

### 5.3 Exogenous ketone supplements

Exogenous ketone supplements mainly consist of ketone esters (KE), ketone salts (KS), 1,3-butanediol, and medium-chain triglycerides (MCT). These agents can rapidly induce mild to moderate ketosis without dietary restriction (131). As the most potent exogenous ketogenic agent currently available, oral KE is hydrolyzed by esterases in the intestine and liver to directly release free BHB or convert into acetyl-CoA, thereby entering the ketone metabolic pathway. However, due to poor palatability, low gastrointestinal tolerance and high costs, KE is currently limited primarily to research applications (132, 133). KS are salt complexes formed by BHB and mineral cations. They dissociate in the gastrointestinal tract and directly release BHB into the circulation, exerting rapid onset within approximately 30–60 minutes, while their ketogenic potency is generally lower than that of KE (134). 1,3-butanediol acts as a ketone precursor. It is progressively oxidized to BHB via hepatic alcohol dehydrogenase and aldehyde dehydrogenase. Its ketogenic effect relies on hepatic oxidative capacity, resulting in a slow, mild and long-lasting elevation of KBs (132). MCT oils are composed of fatty acids of 8 to 12 carbons, mostly octanoate (8:0) and decanoate (10:0). After portal venous absorption and hepatic uptake, MCT undergo rapid β-oxidation to produce acetyl-CoA, which is further converted into KBs. The ketogenic capacity of MCT depends on individual hepatic fatty acid oxidation efficiency, leading to substantial interindividual variation. Accordingly, relatively high doses are usually required to markedly increase circulating BHB levels (135).

Exogenous ketone supplements remain in the exploratory stage in the field of kidney diseases. In mouse models of renal ischemia-reperfusion injury, both preoperative fasting (plasma BHB approximately 2.0 mmol/L) and exogenous BHB infusion (plasma BHB of 1.3–1.4 mmol/L) independently exert renoprotective effects. However, their combined intervention further raised plasma BHB to approximately 4.7 mmol/L without producing additive renal protection. This indicates that the renoprotective action of BHB operates within an optimal concentration range (roughly 1–2 mmol/L), and excessively high concentrations yield no additional benefits (86). Clinically, several early randomized controlled trials are currently underway. For instance, a randomized, double-blind, crossover trial enrolling 14 patients with ADPKD and 29 patients with proteinuric CKD is evaluating the effects of R-1,3-butanediol supplementation on ACR and eGFR after a 4-week intervention, although relevant results have not yet been published.

The major advantages of exogenous ketone supplements include the rapid induction of controllable ketogenesis without dietary restriction, improved treatment adherence, and the avoidance of early KDs-related adverse reactions such as “keto flu”. Nevertheless, this strategy also has notable limitations. The electrolyte load induced by KS may raise safety concerns in patients with CKD. KE are costly and poorly tolerated in terms of taste. In addition, current evidence is predominantly derived from animal studies. Further investigations are therefore warranted to clarify the impacts of exogenous ketone supplements on kidney diseases.

## 6 Discussion

Ketone body metabolism exhibits a functional dichotomy in kidney diseases: BHB exerts renoprotective effects through inhibiting mTORC1, counteracting oxidative stress, epigenetic regulation, and suppressing programmed cell death (54, 55, 79–81, 84–86, 89, 90); whereas HMGCS2-mediated ketogenesis protects the kidney in AKI but may drive inflammation and fibrosis progression in chronic pathological contexts such as DN due to aberrant activation or post-translational modifications (91–97, 101, 102). Therefore, therapeutic strategies targeting ketone body metabolism should pursue “moderate induction” rather than unidirectional activation.

Nevertheless, several outstanding questions remain. The differential regulation of ketone body metabolism across renal cell types is poorly understood. The mechanism behind how HMGCS2 switches from protective to pathogenic remains unknown. Additionally, the long-term safety and efficacy of ketogenic interventions on renal hard endpoints have not been rigorously tested in large RCTs. Furthermore, current knowledge focuses on ADPKD and early DN, while other glomerulopathies remain a virtually blank area. Addressing these gaps will be essential to translate ketone-based strategies into clinical practice.

Future researches should focus on the following directions. First, the generation of cell-type-specific knockout models will be essential to dissect the fine-tuned mechanisms of ketone body metabolism in the kidney. Second, proteomic approaches should be employed to identify regulatory modification sites of HMGCS2, thereby enabling the development of precision intervention strategies. Third, well-designed long-term clinical trials are warranted to clarify the risk-benefit ratio of ketogenic interventions across different stages of CKD. A deeper understanding of the regulatory network of ketone body metabolism will provide new perspectives for metabolic therapy in kidney diseases.

## 7 Conclusion

To sum up, the role of ketone body metabolism in the kidney cannot be ignored in the study of the occurrence and development of kidney diseases and treatment strategies. However, more basic and clinical studies are needed to verify how ketone body metabolism affects different kidney diseases, how much energy ATP produced by its oxidative metabolism can supply kidney cells, and whether KBs are made by the kidney. The KDs and exogenous ketone body supplements are still controversial topics that many medical experts openly support, but there are also many doubts and objections. Currently, metabolic research is a hot spot in the medical field, and experimental and therapeutic research related to ketone body metabolism is also progressing rapidly. Intervention in ketone body metabolism may be an effective means of treating kidney diseases.
